# Supplementary figures and images for: Background Selection as Baseline for Nucleotide Variation across the Drosophila Genome
Source: PLoS Genet. 2014 Jun 26;10(6):e1004434. doi: 10.1371/journal.pgen.1004434 (PMC4072542; doi:10.1371/journal.pgen.1004434)

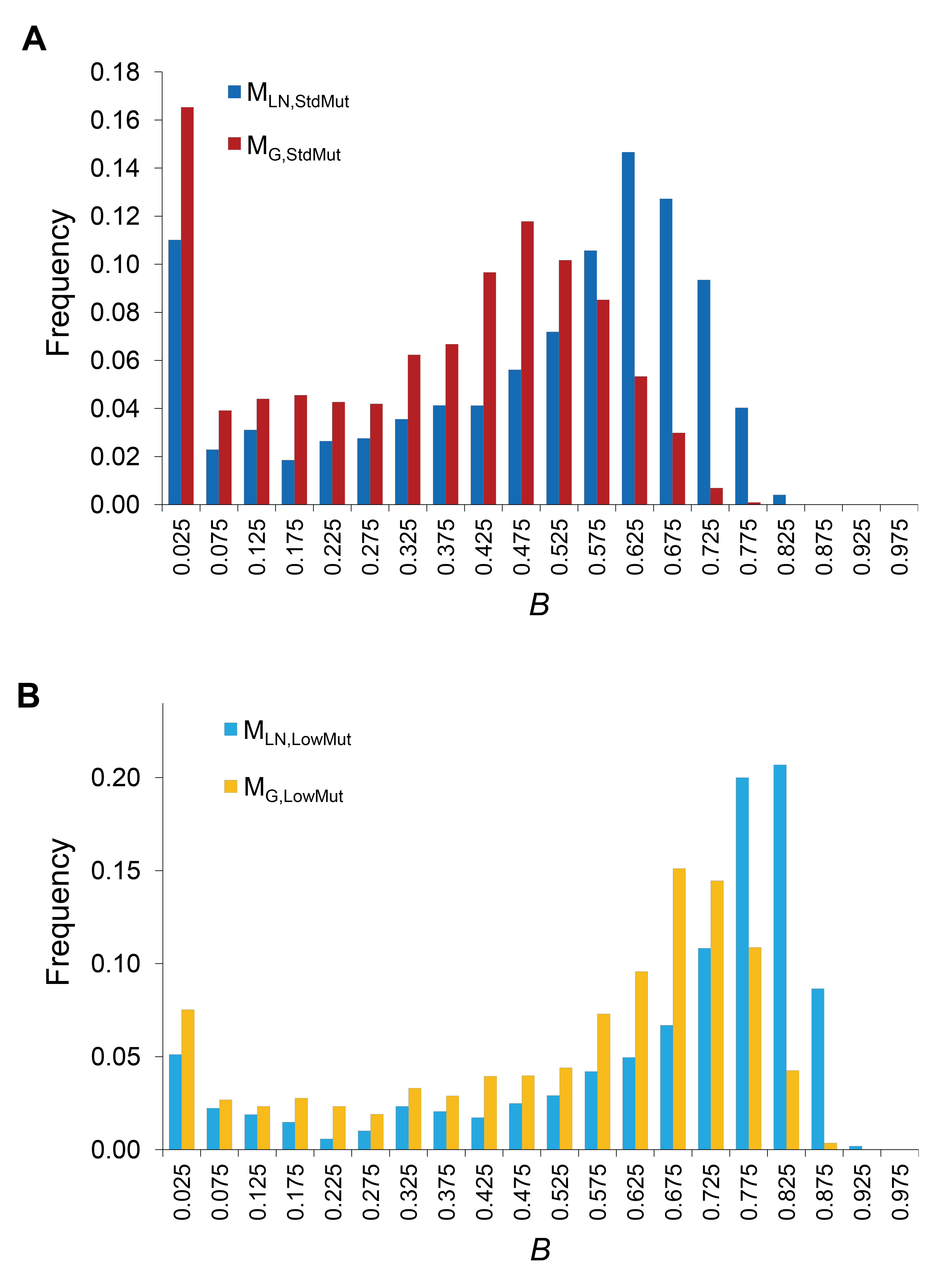

Supplement: Figure S1 — Frequency distribution of estimates of B from BGS models that differ in the distribution of deleterious fitness effects (DDFE) and deleterious mutation rate. (A) Estimates of B based on models MLN,StdMut (log-normal DDFE) and MG,StdMut (gamma DDFE) when the diploid deleterious mutation rate is U = 1.2. (B) Estimates of B based on models MLN,LowMut (log-normal DDFE) and MG,LowMut (gamma DDFE) when the diploid deleterious mutation rate is U = 0.6 (see text for details). All results based on the analysis of 1-kb non-overlapping regions. (TIF) [file pgen.1004434.s001.tif]

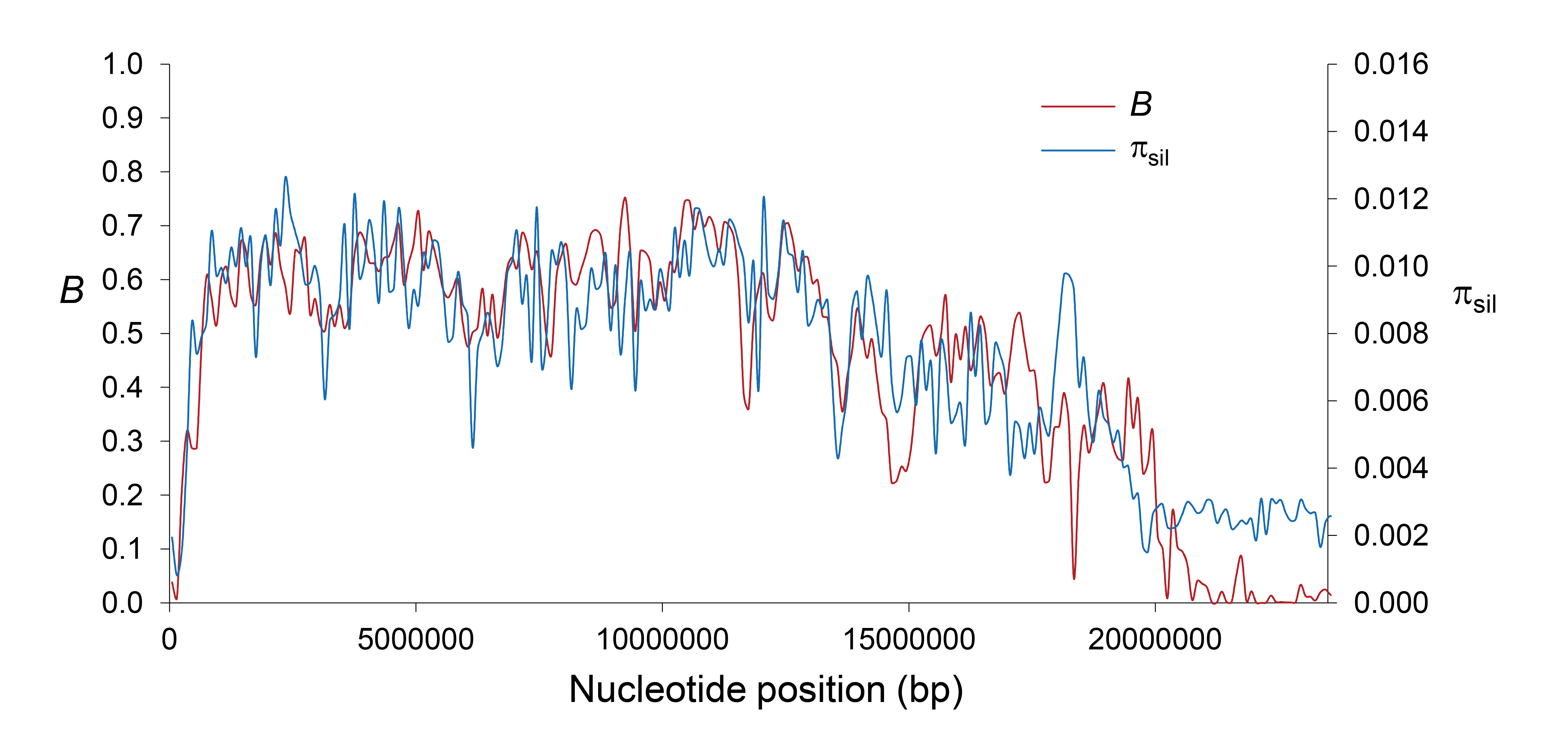

Supplement: Figure S2 — Distribution of silent diversity (πsil) and predicted BGS effects (B). Estimates of B based on model MLN,StdMut,CO+GC. Results shown for 100-kb non-overlapping regions across chromosome arm 3L. (TIF) [file pgen.1004434.s002.tif]
