# Supplementary material for: Background Selection as Baseline for Nucleotide Variation across the Drosophila Genome
Source: PLoS Genet. 2014 Jun 26;10(6):e1004434. doi: 10.1371/journal.pgen.1004434 (PMC4072542; doi:10.1371/journal.pgen.1004434)
Supplement: Table S1 — Summary of B estimates from the different BGS models. (PDF) [file pgen.1004434.s003.pdf]

Table S1. Summary of *B* estimates for different BGS models

		Deleterious Mutation Rate (U = 1.2)				Low Deleterious Mutation Rate (U = 0.6)			
		Log-Normal DDFE		Gamma DDFE		Log-Normal DDFE		Gamma DDFE	
		Rec. CO+GC	Rec. CO	Rec. CO+GC	Rec. CO	Rec. CO+GC	Rec. CO	Rec. CO+GC	Rec. CO
		**$M_{LN,StdMut,CO+GC}$***	$M_{LN,StdMut,CO}$	$M_{G,StdMut,CO+GC}$	$M_{G,StdMut,CO}$	$M_{LN,LowMut,CO+GC}$	$M_{LN,LowMut,CO}$	$M_{G,LowMut,CO+GC}$	$M_{G,LowMut,CO}$
**Whole genome**									
All	Median	**0.591**	0.470	0.428	0.337	0.769	0.686	0.654	0.581
	min, max	**0 , 0.897**	0 , 0.886	0 , 0.870	0 , 0.855	0.005 , 0.947	0.002 , 0.942	0.002 , 0.933	0.001 , 0.925
	90% CI	**0.005 , 0.800**	0 , 0.756	0.001 , 0.707	0 , 0.658	0.074 , 0.895	0.022 , 0.870	0.039 , 0.841	0.011 , 0.811
Autosomes	Median	**0.559**	0.432	0.395	0.303	0.748	0.658	0.628	0.550
	min, max	**0 , 0.822**	0 , 0.804	0 , 0.765	0 , 0.75	0.005 , 0.907	0.002 , 0.897	0.002 , 0.875	0.001 , 0.866
	90% CI	**0.002 , 0.746**	0 , 0.704	0.001 , 0.635	0 , 0.592	0.048 , 0.864	0.017 , 0.839	0.027 , 0.797	0.009 , 0.769
X	Median	**0.736**	0.645	0.603	0.509	0.859	0.804	0.777	0.714
	min, max	**0.025 , 0.897**	0 , 0.886	0.003 , 0.870	0 , 0.855	0.160 , 0.947	0.012 , 0.942	0.056 , 0.933	0.004 , 0.925
	90% CI	**0.075 , 0.862**	0.003 , 0.833	0.013 , 0.810	0.001 , 0.771	0.277 , 0.929	0.057 , 0.913	0.116 , 0.900	0.024 , 0.878
**Trimmed genome**									
All	Median	**0.643**	0.550	0.493	0.416	0.802	0.742	0.702	0.645
	min, max	**0.191 , 0.897**	0.004 , 0.886	0.074 , 0.870	0.006 , 0.855	0.438 , 0.947	0.060 , 0.942	0.271 , 0.933	0.077 , 0.925
	90% CI	**0.411 , 0.814**	0.218 , 0.776	0.238 , 0.730	0.137 , 0.684	0.641 , 0.902	0.467 , 0.881	0.488 , 0.854	0.370 , 0.827
Autosomes	Median	**0.619**	0.517	0.467	0.386	0.787	0.719	0.683	0.621
	min, max	**0.191 , 0.822**	0.004 , 0.804	0.074 , 0.765	0.006 , 0.75	0.438 , 0.907	0.060 , 0.897	0.271 , 0.875	0.077 , 0.866
	90% CI	**0.392 , 0.757**	0.196 , 0.719	0.223 , 0.654	0.12 , 0.615	0.626 , 0.870	0.443 , 0.848	0.472 , 0.809	0.346 , 0.784
X	Median	**0.761**	0.683	0.641	0.561	0.873	0.827	0.801	0.749
	min, max	**0.471 , 0.897**	0.147 , 0.886	0.226 , 0.870	0.091 , 0.855	0.687 , 0.947	0.385 , 0.942	0.476 , 0.933	0.302 , 0.925
	90% CI	**0.576 , 0.866**	0.386 , 0.838	0.388 , 0.819	0.249 , 0.781	0.760 , 0.931	0.623 , 0.916	0.623 , 0.905	0.500 , 0.884

* Model $M_{LN,StdMut,CO+GC}$ is the default model.
