# Supplementary material for: Background Selection as Baseline for Nucleotide Variation across the Drosophila Genome
Source: PLoS Genet. 2014 Jun 26;10(6):e1004434. doi: 10.1371/journal.pgen.1004434 (PMC4072542; doi:10.1371/journal.pgen.1004434)
Supplement: Table S3 — Pairwise Spearman's rank correlation coefficients (ρ) between estimates of B from different BGS models. (PDF) [file pgen.1004434.s005.pdf]

Table S3. Pairwise Spearman's rank correlation coefficients (*ρ*) between estimates of *B* from different BGS models*

	$M_{LN,CO+GC,StdMut}$	$M_{LN,CO,StdMut}$	$M_{G,CO+GC,StdMut}$	$M_{G,CO,StdMut}$	$M_{LN,CO+GC,LowMut}$	$M_{LN,CO,LowMut}$	$M_{G,CO+GC,LowMut}$
$M_{LN,CO,StdMut}$	0.98784						
$M_{G,CO+GC,StdMut}$	0.99792	0.98569					
$M_{G,CO,StdMut}$	0.99132	0.99717	0.99303				
$M_{LN,CO+GC,LowMut}$	0.99999	0.98760	0.99782	0.99105			
$M_{LN,CO,LowMut}$	0.98798	0.99998	0.98579	0.99713	0.98775		
$M_{G,CO+GC,LowMut}$	0.99796	0.98560	0.99999	0.99293	0.99786	0.98570	
$M_{G,CO,LowMut}$	0.99141	0.99715	0.99310	0.99999	0.99114	0.99714	0.99300

* See Materials and Methods for detailed description of the eight BGS models. $P < 1x10^{-12}$ in all cases.
